# Accessing Forbidden Glass Regimes through High-Pressure Sub-*T*_g_ Annealing

**DOI:** 10.1038/srep46631

**Published:** 2017-04-18

**Authors:** Mouritz N. Svenson, John C. Mauro, Sylwester J. Rzoska, Michal Bockowski, Morten M. Smedskjaer

**Affiliations:** 1 Department of Chemistry and Bioscience, Aalborg University, Aalborg 9220, Denmark; 2 Science and Technology Division, Corning Incorporated, Corning, NY 14831, USA; 3 Institute of High Pressure Physics, Polish Academy of Sciences, Warsaw 00-142, Poland

## Abstract

Density and hardness of glasses are known to increase upon both compression at the glass transition temperature (*T*_g_) and ambient pressure sub-*T*_g_ annealing. However, a serial combination of the two methods does not result in higher density and hardness, since the effect of compression is countered by subsequent annealing and vice versa. In this study, we circumvent this by introducing a novel treatment protocol that enables the preparation of high-density, high-hardness bulk aluminosilicate glasses. This is done by first compressing a sodium-magnesium aluminosilicate glass at 1 GPa at *T*_g_, followed by sub-*T*_g_ annealing *in-situ* at 1 GPa. Through density, hardness, and heat capacity measurements, we demonstrate that the effects of hot compression and sub-*T*_g_ annealing can be combined to access a “forbidden glass” regime that is inaccessible through thermal history or pressure history variation alone. We also study the relaxation behavior of the densified samples during subsequent ambient pressure sub-*T*_g_ annealing. Density and hardness are found to relax and approach their ambient condition values upon annealing, but the difference in relaxation time of density and hardness, which is usually observed for hot compressed glasses, vanishes for samples previously subjected to high-pressure sub-*T*_g_ annealing. This confirms the unique configurational state of these glasses.

Functional glasses with tailored properties are expected to play a critical role in a range of developing technologies^1^, and there is thus a need for inventing new methods to tune the glass properties. In particular, the mechanical properties of glass have received wide attention, since the brittleness and low practical strength of oxide glasses are major bottlenecks for future applications^1^. Various methods for improving the damage resistance of glasses have been attempted, including composition design, thermal tempering, surface crystallization, and chemical strengthening^2^. However, development of new methods to prepare damage resistant glasses is desired to push the limits of their applications, but the understanding of structure-mechanical property relations remains a challenging problem^3^.

An alternative method for modifying the glass structure and properties is to permanently densify the glass, e.g., by subjecting it to isostatic compression at *T*_g_ (so-called hot compression), thus changing its fictive pressure^4^,^5^,^6^,^7^ and resulting in increased density and hardness^8^,^9^,^10^,^11^,^12^. Most literature studies have focused on the effects of composition and thermal history on the mechanical properties of glass^13^,^14^,^15^, but pressure can be used as an additional degree of freedom or design parameter to tailor and understand the glass structure-property relations. For example, fundamental relations between volume densification and changes in glass properties are emerging from studies of hot compressed glasses^16^,^17^. The changes in properties induced by densification have been found to depend on the densification method. For example, a recent study of vitreous SiO_2_ subjected to both hot and cold compression has shown that glasses with similar density increase exhibit different intermediate-range order and elastic moduli^18^. Fundamentally different structural changes have also been found to occur in hot- and cold compressed borosilicate glass using *in-situ* high-pressure ^11^B nuclear magnetic resonance (NMR) spectroscopy^19^. The density dependence of structure^20^, hardness^21^, and elastic moduli^16^ has also been found to differ between hot compressed glasses and thermally annealed glasses, i.e., glasses annealed below the initial fictive temperature (so-called sub-*T*_g_ annealing). Recent molecular dynamics simulations have suggested that hot compression mainly affects the intermediate-range order of an aluminosilicate glass, whereas sub-*T*_g_ annealing mainly affects the short-range order^21^.

Since sub-*T*_g_ annealing and hot compression can both be applied to increase the hardness of bulk glasses, it would be desirable if these treatments could be combined to enable the preparation of super-hard glasses. This would be important as hardness, which measures the resistance to elastoplastic deformation, is an important mechanical property of glasses for applications such as scratch-resistant display covers. However, combining sub-*T*_g_ annealing and hot compression in series (i.e., first subjecting glass to sub-*T*_g_ annealing and then to hot compression or vice versa) is ineffective, since structural changes invoked by the first treatment will be countered by that of the second one. For example, ambient pressure sub-*T*_g_ annealing of compressed glasses causes relaxation of the pressure-induced effects^9^,^11^,^22^ i.e., the hardness of the glass is not efficiently increased by first performing hot compression and then ambient pressure sub-*T*_g_ annealing.

To circumvent this problem, here we introduce a novel treatment protocol that enables the preparation of high-density, high-hardness bulk aluminosilicate glasses. This is done by first performing 1 GPa compression at *T*_g_, followed by sub-*T*_g_ annealing *in-situ* at 1 GPa. We use a specially designed gas pressure chamber first to perform conventional hot compression (*P* = 1 GPa, *T* = *T*_g_) of a sodium-magnesium aluminosilicate glass, immediately followed by high-pressure *in-situ* sub-*T*_g_ annealing (*P* = 1 GPa, *T* = 0.9 *T*_g_). Sub-*T*_g_ annealing is performed *in-situ* at 1 GPa to avoid pressure relaxation effects that would otherwise occur during ambient pressure annealing^9^,^11^. Our results demonstrate that 1 GPa compression at *T*_g_ can be combined with 1 GPa sub-*T*_g_ annealing to produce aluminosilicate glasses with increased hardness and density that could not have been achieved through thermal history or pressure history variation alone.

The combined effects of variations in the pressure and temperature path during glass formation on structure and properties are not well understood. Changes in cooling rate have been shown to have similar effect on boron speciation at ambient pressure and 0.5 GPa for a borosilicate glass^12^. However, for two aluminosilicate glasses quenched from liquid state at 10 GPa or annealed near *T*_g_ at 10 GPa, the lower compression temperature caused an increase in Al coordination in fully polymerized albite composition, but a decrease in a depolymerized composition^23^. Previous work has also shown that the structure and properties of hot compressed glasses can be relaxed during ambient pressure sub-*T*_g_ annealing^24^,^25^. For a deeper understanding of the relaxation behavior of compressed glasses, we also study the changes in density, hardness, and heat capacity of our glasses during ambient pressure sub-*T*_g_ annealing.

## Methods

**Figure 1:**
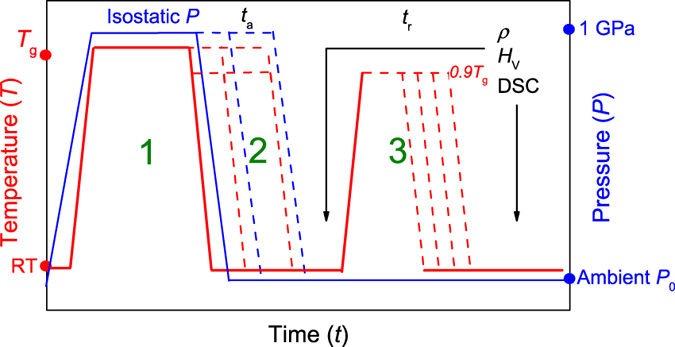
Overview of experimental design. Step 1: Temperature and pressure is raised to *T*_g_ and 1 GPa, respectively. The sample is kept under these conditions for 30 min. Step 2: Additional *in-situ* annealing at 1 GPa was done at either *T*_g_ or 0.9 *T*_g_ for durations (*t*_a_) up to 24 h. After annealing, the temperature was decreased to room temperature, followed by decompression. Step 3: Both compressed and compressed/sub-*T*_*g*_ annealed samples were subject to ambient pressure annealing at 0.9 *T*_g_ for various durations (*t*_r_). Density (*ρ*), hardness (*H*_V_), and differential scanning calorimetry (DSC) measurements were performed following each of the three steps.

The glass used in this study is a commercial sodium-magnesium aluminosilicate glass^26^, identical to the one used in two recent studies^20^,^27^. The glass was prepared by the fusion draw method, resulting in high fictive temperature and excellent surface quality making it well suited for indentation experiments. Samples (2.5 cm × 2.5 cm) were then subjected to different hot compression and sub-*T*_g_ annealing treatments, as explained in the following and illustrated in Fig. 1.

Hot compression of the samples was performed using a nitrogen gas pressure chamber, described in detail elsewhere^28^. The setup consists of a multizone cylindrical furnace, which is placed inside a gas pressure reactor with nitrogen as the compression medium. During compression, the samples were heated at a rate of 600 K/h up to their ambient pressure *T*_g_ value (652 °C), with a simultaneous pressure increase up to 1 GPa (we note that the glass transition temperature changes as a function of pressure^29^, but the change is expected to be small within this pressure regime^30^). As a first step, all compressed samples were kept under these conditions for 30 min. Following this step, one set of samples were cooled to room temperature at a rate of 60 K/min, followed by decompression at a rate of 30 MPa/min (step 1 in Fig. 1). Another set of samples were only cooled to 0.9 *T*_g_ (560 °C), while the pressure remained constant at 1 GPa. These samples were kept under these conditions for 2 or 24 h, followed by quenching to ambient conditions (step 2 in Fig. 1). To compare the effect of compression at 0.9 *T*_*g*_ with compression at *T*_*g*_, another set of samples were compressed at *T*_g_ at 1 GPa for 2 and 24 h. To compare the effect of sub-*T*_*g*_ annealing at 1 GPa with sub-*T*_*g*_ annealing at ambient pressure, a series of pristine samples were subjected to annealing at 0.9 *T*_g_ at ambient pressure for 2 h and 24 h. Following the above described treatments, all samples were subjected to relaxation experiments by ambient pressure annealing at 0.9 *T*_g_ for 23500 min (~16 days) (step 3 in Fig. 1).

Selected samples were characterized after the various treatments by density measurements, differential scanning calorimetry (DSC), and Vickers microindentation. Density measurements were performed using Archimedes method, with ethanol as the auxiliary liquid. Vickers hardness (*H*_V_) was determined using a Duramin 5 microindenter (Struers A/S), with load and holding time of 0.1 N and 10 s, respectively. Compression is known to modify the glass transition behavior^9^. However, changes in the glass transition behavior of compressed glasses have not previously been investigated during relaxation. Such information may help to clarify fundamental questions about the relaxation behavior, e.g., whether the compressed glass relaxes towards the prior as-prepared state, or another energy state. DSC measurements were therefore performed on samples before compression, after compression, and throughout relaxation, using a simultaneous thermal analysis instrument (STA 449 F1 Jupiter, Netzsch). In order to determine the isobaric heat capacity (*C*_p_) as a function of temperature at ambient pressure, a baseline measurement was performed using two empty Pt/Rh crucibles, followed by a calibration measurement using a standard reference (sapphire) with known heat capacity. Scan rates of 10 K/min were applied during each up and down scan. From the heat capacity curves, various parameters characterizing the glass transition were quantified. The onset temperature for the glass transition region (*T*_g,onset_) was determined as the intercept between the tangent of the *C*_p_(*T*) slope before the glass transition and the tangent to the inflection point during the glass transition. The offset of the glass transition region (*T*_g,offset_) was determined from the intercept of the tangents in the inflection point (on high temperature side of overshoot) and the heat capacity of the supercooled liquid. The width of the glass transition region (Δ*T*_g_) was determined as the difference between *T*_g,onset_ and *T*_g,offset_. The overshoot in heat capacity during glass transition (Δ*H*_overshoot_) was determined from the area of the *C*_p_ curve above the supercooled liquid line.

## Results and Discussion

### Combining sub-*T*_g_ annealing and hot compression

**Figure 2:**
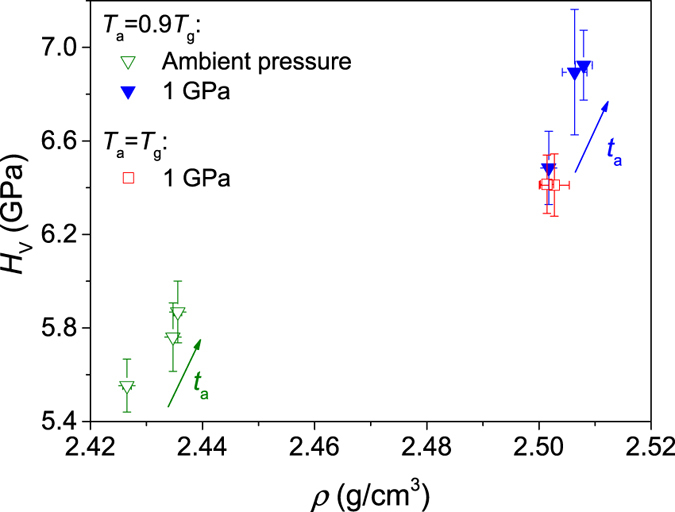
Dependence of Vickers hardness (*H*_V_) on density (*ρ*) for glasses subjected to three different treatments. 0.9 *T*_g_ annealing at ambient pressure (open green triangles), 1 GPa compression at *T*_g_ for durations of 2 or 24 h (red open squares, step 1 and 2 in Fig. 1), and 1 GPa compression at *T*_g_ followed by 0.9 *T*_g_ annealing at 1 GPa for 0, 2, or 24 h (solid blue triangles, step 2 in Fig. 1). The inserted arrows denote increasing sub-*T*_g_ annealing duration (*t*_a_).

Figure 2 shows the dependence of Vickers hardness on density for the samples subjected to 0.9 *T*_g_ annealing at ambient pressure, 1 GPa compression at *T*_g_ for durations up to 24 h (step 1 and 2 in Fig. 1), and 1 GPa compression at *T*_g_ followed by 0.9 *T*_g_ annealing at 1 GPa (step 2 in Fig. 1). Hardness and density increase with sub-*T*_g_ annealing time, both at ambient pressure and 1 GPa. These properties do not change significantly with compression time at 1 GPa and *T*_g_, This shows that the increase in density and hardness after compression at 0.9 *T*_g_ at 1 GPa is not a result of prolonged compression duration alone, but also a result of the temperature applied during compression. We note that by combining hot compression and high-pressure sub-*T*_g_ annealing, it is possible to prepare harder glasses than through hot compression or sub-*T*_g_ annealing alone.

**Figure 3:**
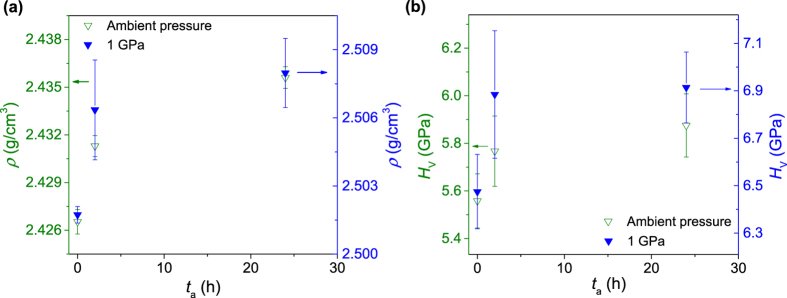
(**a**) Density *ρ* and (**b**) Vickers hardness *H*_V_ as a function of the sub-*T*_g_ annealing duration *t*_a_ at ambient pressure and *in-situ* at 1 GPa. In both figures, the range of density and hardness values covered is identical on both primary and secondary vertical, but the absolute values are offset for clarity. The effect of annealing on both density and hardness is similar both at ambient pressure and at 1 GPa. It should be noted that the samples with *t*_a_ = 0 h exhibit different values of density and hardness, since the “1 GPa” sample has been hot compressed without subsequent *in-situ* sub-*T*_g_ annealing, whereas the “ambient pressure” sample has not been subject to any compression.

**Figure 4:**
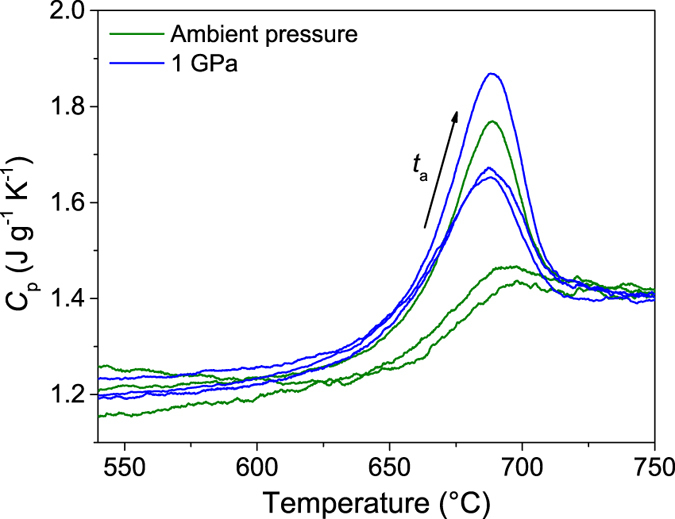
Heat capacity (*C*_p_) vs. temperature curves in the glass transition range for the glasses subjected to 0.9 *T*_g_ annealing at ambient pressure and *in-situ* at 1 GPa. The inserted arrow denotes increasing sub-*T*_g_ annealing duration (*t*_a_) equal to 0, 2, or 24 h.

Next we compare sub-*T*_g_ annealing under ambient and 1 GPa pressures. The density and hardness are shown as a function of annealing duration in Fig. 3a and b, respectively. Within the experimental uncertainty, the changes in density and hardness exhibit similar time dependence, independent of the pressure applied during sub-*T*_g_ annealing. This pressure-independent behavior is comparable with earlier findings that the cooling rate dependence of boron coordination and fictive pressure is similar at ambient pressure and at 0.5 GPa^12^. Figure 4 shows the effect of sub-*T*_g_ annealing at ambient and 1 GPa pressure on the calorimetric glass transition. The enthalpy overshoot (Δ*H*_overshoot_) is the area of the heat capacity curve above the supercooled liquid line. As seen from the figure, Δ*H*_overshoot_ increases upon sub-*T*_g_ annealing at ambient pressure, i.e., upon decreasing fictive temperature, as it has previously been observed for a variety of as-prepared glasses^31^,^32^,^33^. Moreover, hot compression causes an increase in the enthalpy overshoot, as also previously found for related glass compositions^9^,^10^. The degree of overshoot increases further after compression at 0.9 *T*_g_ at 1 GPa. Sub-*T*_*g*_ annealing for 24 h increases the Δ*H*_overshoot_ by a similar magnitude at both ambient and 1 GPa pressure, indicating a similar effect of sub-*T*_g_ annealing on Δ*H*_overshoot_ at the two pressures.

The abovementioned changes in density and hardness (Fig. 3) and glass transition behavior (Fig. 4) as a function of sub-*T*_g_ annealing time at different pressures indicate that the effect of sub-*T*_g_ annealing is equivalent at the two pressures (ambient and 1 GPa). This shows that it is indeed possible to combine the structural transformations induced by isostatic compression at *T*_g_ and sub-*T*_g_ annealing to produce a glass with increased hardness and density. As such, a modification of the energy landscape can be obtained by combining the effects of thermal annealing and hot compression, since the energy landscape is a complicated function of pressure. The treatment thus expands the region of phase space accessible to the glass, i.e., it enables access to a so-called “forbidden glass” regime following the terminology of Mauro and Loucks^34^, which is a regime that is inaccessible through thermal history or pressure history variation alone.

### Pressure relaxation

**Figure 5:**
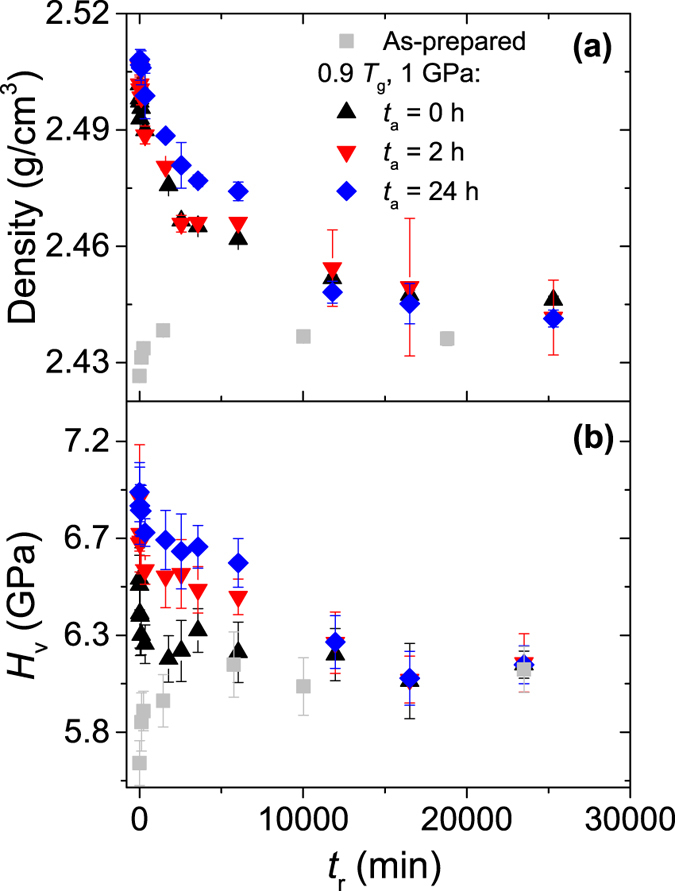
Dependence of (**a**) hardness and (**b**) density on the duration (*t*_r_) of the 0.9 *T*_g_ ambient pressure annealing (i.e., step 3 in Fig. 1). Results are shown for as-prepared glasses and glasses subjected to *in-situ* high-pressure sub-*T*_g_ annealing for different durations (*t*_a_) prior to relaxation (i.e., steps 1 and 2 in Fig. 1).

The next question that arises is how stable the properties of the hot compressed glasses are during subsequent ambient pressure annealing (relaxation). Figure 5 shows the relaxation behavior of Vickers hardness and density during 0.9 *T*_g_ annealing at ambient pressure, for as-prepared glasses and hot compressed glasses. Similarly to previous findings^9^,^10^,^11^, the hardness and density of the hot compressed glasses are found to decrease during ambient pressure annealing. The density and hardness of the as-prepared glass increase during annealing due to relaxation. After prolonged ambient pressure annealing (~10,000 min), density and hardness for all samples are found to converge towards the same values, indicating that the samples relax towards the same configurational state. Furthermore, the properties of this relaxed state appear to be governed by the annealing temperature, i.e., complete relaxation of the densified structure occurs at 0.9 *T*_g_.

The samples with different sub-*T*_g_ annealing durations at 1 GPa exhibit different hardness and density values before relaxation, and these differences remain pronounced into the relaxation process (Fig. 5). This can be understood based on the previously observed equivalent effects of sub-*T*_g_ annealing at ambient and 1 GPa pressure (Fig. 3). That is, the effect of sub-*T*_g_ annealing at 1 GPa will not be countered by ambient pressure sub-*T*_g_ annealing, if the effects of both treatments are the same. Throughout sub-*T*_g_ annealing at ambient pressure, the effects of sub-*T*_g_ annealing at 1 GPa will increase, while the effects resulting from pressure relax. After complete pressure relaxation, the effects on hardness and density resulting from sub-*T*_g_ annealing at different pressures remain. This is in agreement with the finding that the compressed and non-compressed samples all relax toward the same state after long term sub-*T*_g_ annealing, i.e., no relaxation occurs beyond this point.

To further analyze the relaxation behavior of hardness and density, we first normalize the values using the following relaxation function *M*_p_(*t*_a_):


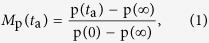


where *M*_p_(*t*_a_) is the fraction of the property (p) relaxed at time *t*_a_ and p(0), p(*t*_a_), and p(∞) are the values of the property (hardness or density) before annealing, at the given annealing time step *t*_a_, and of the uncompressed sample after infinite annealing time, respectively. Since infinite annealing time reaches beyond the timescale of our laboratory experiments, we use the hardness or density value of the uncompressed sample stabilized after prolonged annealing (e.g., >10,000 min) as p(∞). The relaxation function can then be fitted with the Kohlrausch stretched exponential function^35^:


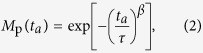


where *τ* is the characteristic relaxation time for the decay and *β* is the dimensionless stretching exponent. *β* varies between 1 (corresponding to simple exponential decay) and 0. It has previously been suggested that *β* will be fixed at one of two universal values in the limit of temperatures at or below *T*_g_. That is, *β* = 3/5 for relaxation processes involving both short- and long-range rearrangements, and *β* = 3/7 for relaxation dominated by long-range rearrangements^36^.

**Figure 6:**
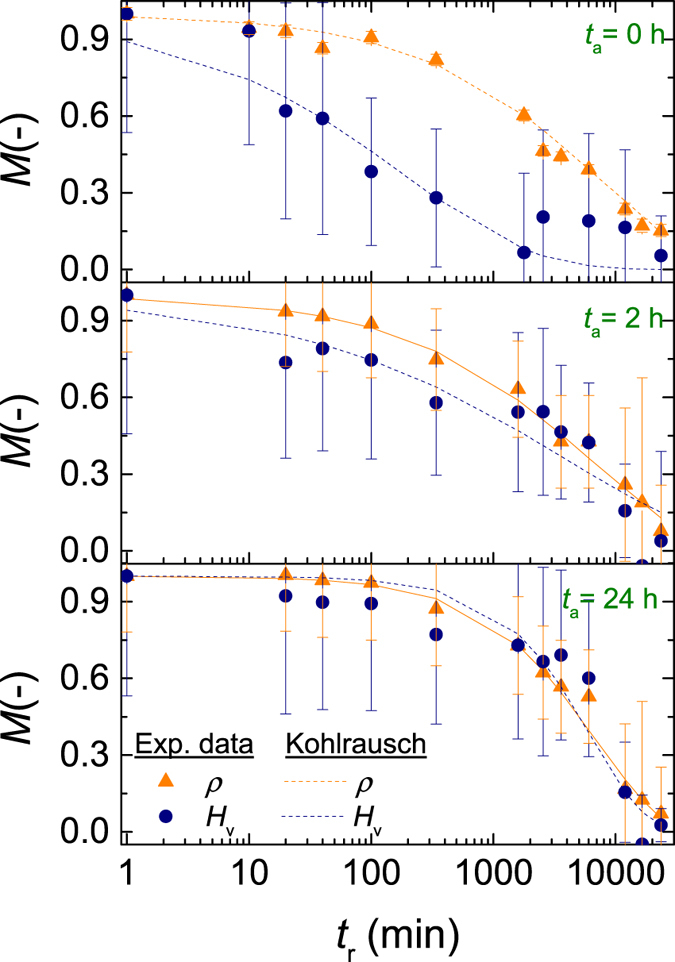
Annealing (relaxation) time (*t*_r_) dependence of the relaxation function (*M*) for density and Vickers hardness throughout ambient pressure annealing at 0.9 *T*_g_. Results are shown for glasses subjected to *in-situ* high-pressure sub-*T*_g_ annealing for different durations (*t*_a_). The dashed lines represent fits to Eq. (2).

Figure 6 shows the relaxation function and stretched exponential fit for the compressed glasses. During hardness and density relaxation of hot compressed glasses, it has previously been observed that hardness relaxes on a shorter time scale than density^9^,^11^. This is indeed observed in Fig. 6 for the samples subjected to 1 GPa compression at *T*_g_ (step 1 in Fig. 1), thus agreeing with the previous results. However, for the samples also subjected to *in-situ* high-pressure sub-*T*_g_ annealing (step 2), the timescales of hardness and density relaxation converge. The structural changes induced by sub-*T*_g_ annealing have been suggested to strengthen the network connectivity^21^. When the sub-*T*_g_ annealing is performed at 1 GPa, we expect a similar change in network connectivity, which might inhibit different parts of the glass network from relaxing at different time scales, resulting in coupled relaxation of density and hardness. It is well known that the thermal history of a glass can influence its relaxation behavior. For example, it has been demonstrated that glasses of similar refractive index, produced by annealing at constant temperature or slow cooling, exhibit different relaxation behaviors during subsequent relaxation at constant temperature^37^,^38^. Furthermore, the non-equilibrium viscosity (i.e., relaxation time for plastic flow) at sub-*T*_g_ annealing temperatures has been found to depend on the thermal history of the glass^39^. The pressure relaxation of hardness observed here could be considered to exhibit an analogous behavior, since the relaxation time for hardness depends on the thermal history of the glass (i.e., annealing duration at 0.9 *T*_g_ at 1 GPa) as seen by comparing Fig. 6a,b and c.

**Figure 7:**
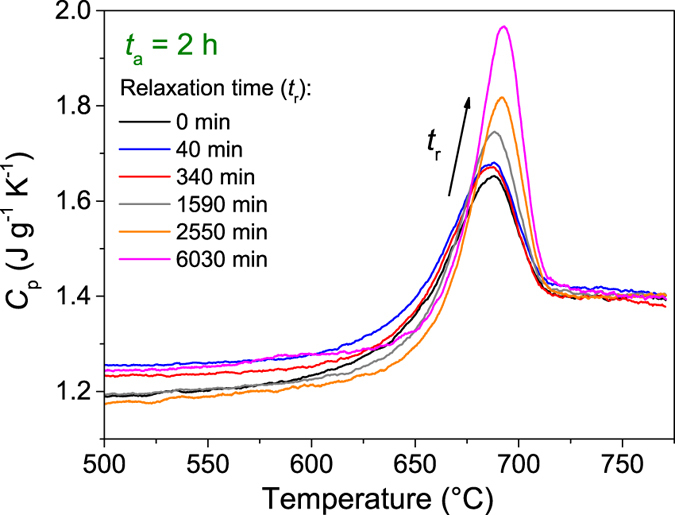
Heat capacity (*C*_p_) vs. temperature curves in the glass transition range for the glasses first hot compressed (step 1), then *in-situ* high-pressure annealed at 0.9 *T*_g_ for *t*_a_ = 2 h (step 2), and finally annealed at 0.9 *T*_g_ at ambient pressure for different durations (step 3). The inserted arrow denotes increasing relaxation duration (*t*_r_).

Figure 7 shows the heat capacity vs. temperature curves in the glass transition region for the sample subjected to sub-*T*_g_ annealing at 1 GPa for *t*_a_ = 2 h and subsequently sub-*T*_g_ annealed at ambient pressure for different durations (*t*_r_). A clear increase in the enthalpy overshoot (Δ*H*_overshoot_) is observed with increasing relaxation time. Similarly, this was also found for the other compressed samples during relaxation (see Figure S1 in the Supplementary Material). The increase in Δ*H*_overshoot_ upon relaxation demonstrates that the compressed samples do not relax towards their prior as-prepared state in terms of the glass transition behavior.

**Figure 8:**
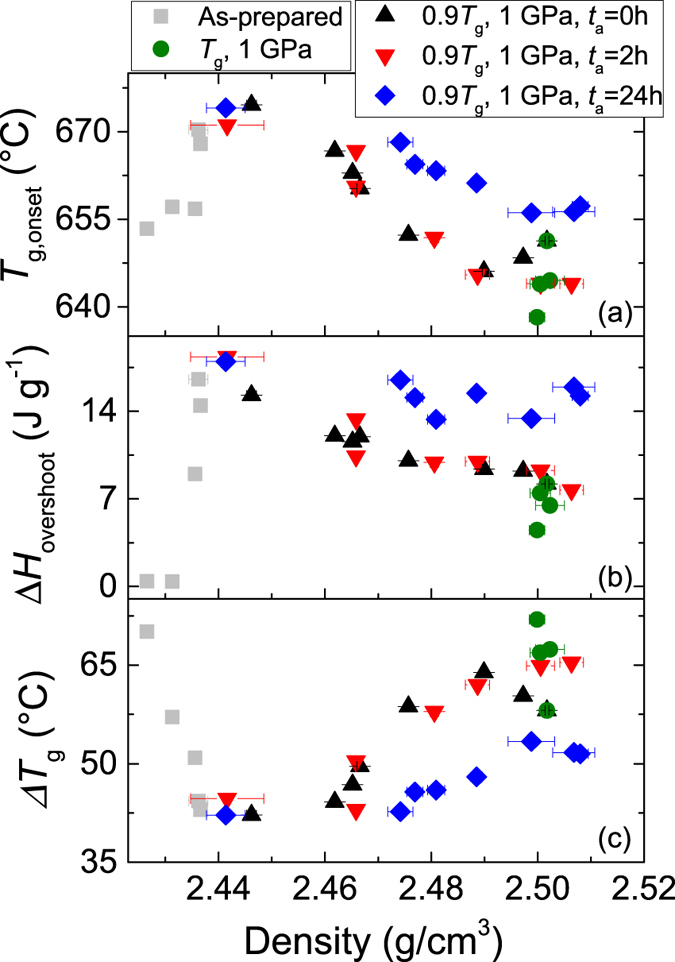
Density (*ρ*) dependence of (**a**) onset temperature of glass transition *T*_g,onset_, (**b**) enthalpy overshoot Δ*H*_overshoot_, and (**c**) width of the glass transition Δ*T*_g_ for samples subjected to ambient pressure 0.9 *T*_g_ annealing performed subsequent to any compression (i.e., step 3 in Fig. 1). Results are shown for as-prepared glasses, glasses hot compressed at 1 GPa for different durations, and glasses also subjected to *in-situ* high-pressure sub-*T*_g_ annealing for different durations (*t*_a_).

The density dependence of *T*_g,onset_, Δ*H*_overshoot_, and width of glass transition (Δ*T*_g_) are shown in Fig. 8a,b and c, respectively, for all the samples throughout relaxation (i.e., step 3 in Fig. 1). Hot compression at *T*_g_ is found to cause clear changes in the values of *T*_g,onset_ and Δ*H*_overshoot_, but only minor changes in Δ*T*_*g*_. High-pressure sub-*T*_g_ annealing for *t*_a_ = 24 h further modifies these properties, and the changes remain pronounced during relaxation. In contrast, no significant effect of sub-*T*_g_ annealing for *t*_a_ = 2 h is seen, when comparing with the *t*_a_ = 0 samples. Following prolonged relaxation, the measured glass transition parameters converge towards approximately the same values for all glass samples (see Figure S2 in the Supplementary Material). This agrees with the relaxation behavior of hardness and density (Fig. 5). That is, ambient pressure sub-*T*_g_ annealing reverses the pressure-induced changes in density, hardness and *T*_g,onset_, showing that the pressure-induced changes in these properties do not “survive” relaxation. The pressure-induced changes in Δ*H*_overshoot_ are found to increase during relaxation, i.e., the thermally relaxed glasses are more stable. Moreover, the Δ*H*_overshoot_ values of all the long-term relaxed glasses are similar, independent of their prior state, which may in turn indicate that all glasses have relaxed to the same enthalpic state.

The parameters *T*_g,onset_, Δ*H*_overshoot_ and Δ*T*_g_ are difficult to interpret unambiguously in terms of their structural origin or relation to other glass properties. Δ*T*_g_ has previously been correlated with the liquid fragility (*m*) of glass-forming liquids^40^,^41^, which describes the extent of non-Arrhenius scaling of viscosity with temperature^42^. A higher value of *m* (“fragile” liquid) leads to a sharper breakdown of ergodicity and a more well-defined glass transition, i.e., Δ*T*_g_ is inversely correlated with *m*^43^. For compressed glasses, we generally find an increase in Δ*T*_g_ with increasing density (Fig. 8c). Such lower fragility at high density could be explained by a lower entropic contribution to dynamics as there are fewer transition states available when the atomic packing density is high^44^,^45^. However, as seen in Fig. 8, the same Δ*T*_g_ can be achieved across various densities for the same glass composition, showing that the relation between density, entropy and Δ*T*_g_ could be more complex.

A similar complexity applies for changes in the *T*_g,onset_ values. After hot compression, the glasses exhibit an overall decrease in *T*_g,onset_ (Fig. 8a). Upon heating from below to above the glass transition, the configuration space converts from a partitioned set of metabasins with slow interbasin transitions to an ergodic state with fast intra- and interbasin transitions^46^,^47^. Less thermal energy is required to drive this change when the network is more tightly packed. However, for other hot compressed glasses, both an increased^12^ and a constant^9^,^48^ *T*_g,onset_ has previously been found with increasing density. For the non-compressed samples, an increase in *T*_g,onset_ is found with increasing density (Fig. 8a). This is similar to previous findings for various non-compressed and sub-*T*_*g*_ annealed inorganic glasses^32^,^33^ and a vapor deposited organic glass^49^. In addition, Fig. 8 shows that the same values of *T*_g,onset_ can be found for various density values. Figure 8 also shows that there is no clear correlation between Δ*H*_overshoot_ and density when considering all the samples. However, during relaxation of the hot compressed samples, we observe an approximate decrease in Δ*H*_overshoot_ with increasing density. Since the densification induced by this treatment gives rise to glasses with higher enthalpy, the enthalpy release at the glass transition gets larger as the density decreases.

It has previously been suggested that hot compression and ambient pressure sub-*T*_g_ annealing increases the hardness and density of aluminosilicate glasses through different structural mechanisms^21^. This difference in structural mechanisms can be confirmed to also apply at 1 GPa, by comparing the samples annealed at 1 GPa for 24 h at either *T*_g_ or 0.9 *T*_g_. Here substantial differences in density, hardness, and glass transition behavior are observed. This suggests that the effect of 0.9 *T*_g_ annealing under 1 GPa pressure on the glass structure and properties is different from that of annealing at *T*_g_ at 1 GPa, in turn indicating that hot compression at *T*_g_ and 0.9 *T*_*g*_ operate through different structural mechanisms.

## Conclusions

Hot isostatic compression (1 GPa at *T*_g_) of a commercial sodium-magnesium aluminosilicate glass causes an increase in density, hardness, and enthalpy overshoot. By combining hot compression with further high pressure *in-situ* sub-*T*_g_ annealing (1 GPa at 0.9 *T*_g_) a further increase in density, hardness and enthalpy overshoot is achieved. The magnitudes of these increases are similar to that obtained by ambient pressure sub-*T*_g_ annealing of the pristine glass. Furthermore, the changes in density and hardness invoked by *in-situ* sub-*T*_g_ annealing (1 GPa at 0.9 *T*_g_) remained pronounced during subsequent relaxation (ambient pressure sub-*T*_g_ annealing). Upon prolonged relaxation of compressed-annealed samples, the onset temperature of glass transition, enthalpy overshoot, and width of the calorimetric glass transition approach similar values independent of the state of the glass prior to relaxation.

## Additional Information

**How to cite this article**: Svenson, M. N. *et al*. Accessing Forbidden Glass Regimes through High-Pressure Sub-*T*_g_ Annealing. *Sci. Rep.* **7**, 46631; doi: 10.1038/srep46631 (2017).

**Publisher's note:** Springer Nature remains neutral with regard to jurisdictional claims in published maps and institutional affiliations.

## Supplementary Material

Supplementary Material
