# Supplementary material for: Accessing Forbidden Glass Regimes through High-Pressure Sub-Tg Annealing
Source: Sci Rep. 2017 Apr 18;7:46631. doi: 10.1038/srep46631 (PMC5394531; doi:10.1038/srep46631)
Supplement: Supplementary Material [file srep46631-s1.pdf]

Supplementary Material

Accessing Forbidden Glass Regimes through High-Pressure Sub-T_g Annealing

Mouritz N. Svenson[a], John C. Mauro[b], Sylwester J. Rzoska[c], Michal Bockowski[c], Morten M. Smedskjaer[a,*]

[a]*Department of Chemistry and Bioscience, Aalborg University, Aalborg 9220, Denmark*

[b]*Science and Technology Division, Corning Incorporated, Corning, NY 14831, USA*

[c]*Institute of High Pressure Physics, Polish Academy of Sciences, Warsaw 00-142, Poland*

[*]*Corresponding author. e-mail: mos@bio.aau.dk*

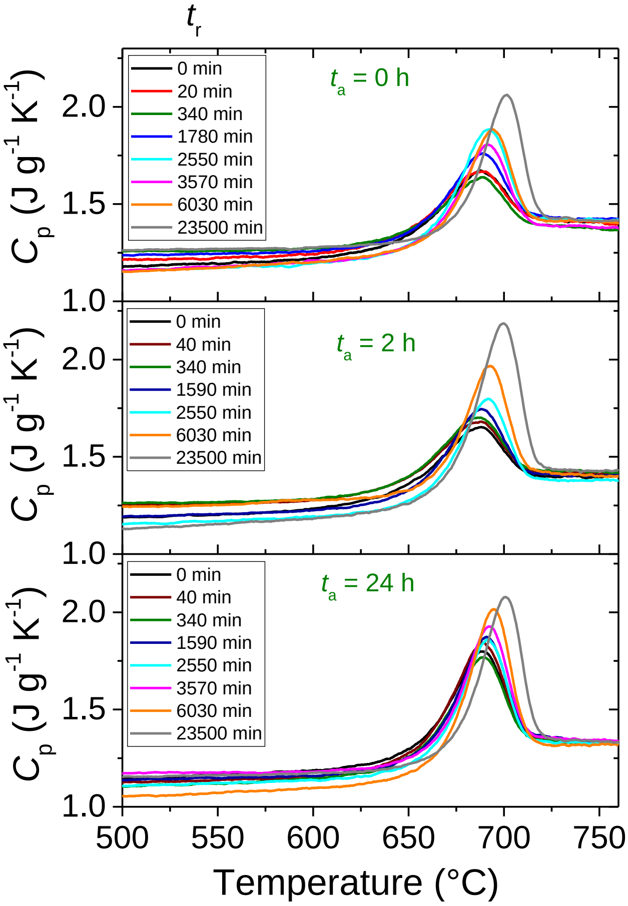

Figure S1. Calorimetric glass transition determined by differential scanning calorimetry of 1 GPa compressed samples subjected to ambient pressure relaxation ($0.9T_g$) for different durations (t_r). Top: samples compressed at T_g before relaxation (step 1 in Figure 1). Middle: samples compressed for t_a = 2 h at $0.9T_g$ prior to relaxation (step 2 in Figure 1). Bottom: samples compressed for t_a = 24 h at $0.9T_g$ prior to relaxation (step 2 in Figure 1).

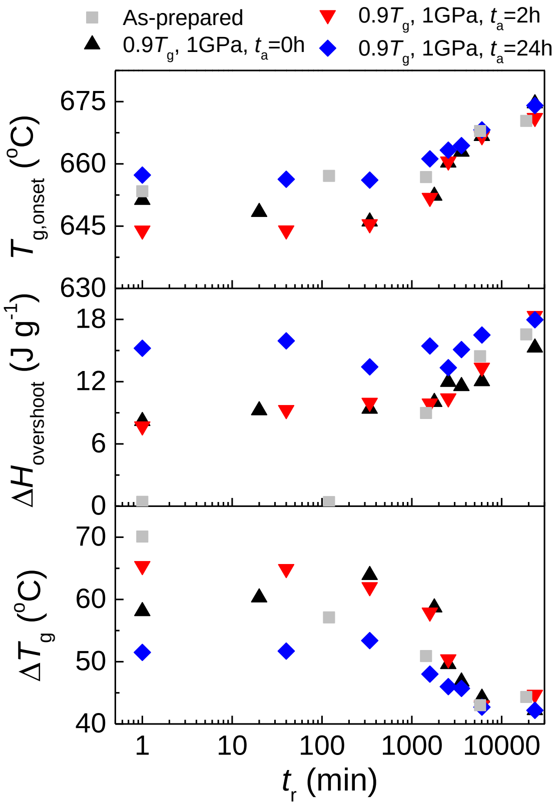

Figure S2. Relaxation time (t_r) dependence of (a) onset temperature of glass transition $T_{g,onset}$, (b) enthalpy overshoot $\Delta H_{overshoot}$, and (c) width of the glass transition ΔT_g for samples subjected to ambient pressure $0.9T_g$ annealing performed subsequent to any compression (i.e., step 3 in Fig. 1). Results are shown for as-prepared glasses and glasses also subjected to *in-situ* high-pressure sub-T_g annealing for different durations (t_a).
